# Micromechanics and Ultrasonic Propagation in Consolidated Earthen-Site Soils

**DOI:** 10.3390/ma16227117

**Published:** 2023-11-10

**Authors:** Yingmin Zhang, Guang Yang, Dongxu Liu, Wenwu Chen, Lizhi Sun

**Affiliations:** 1 CCCC First Harbor Consultants Co., Ltd., Tianjin 300222, China; zhangym16@outlook.com; 2 Department of Civil & Environmental Engineering, University of California, Irvine, CA 92697, USA; dongxul@uci.edu; 3 China Water Resources Beifang Investigation, Design and Research Co., Ltd., Tianjin 300222, China; ldyangguang@126.com; 4 Key Laboratory of Mechanics on Disaster & Environment in Western China, School of Civil Engineering & Mechanics, Lanzhou University, Lanzhou 730000, China; sungp@lzu.edu.cn

**Keywords:** consolidated earthen-site soils, microstructure, micromechanics, finite-element method, wave propagation

## Abstract

Although nondestructive ultrasonic technologies have been applied in laboratory and field tests in the field of heritage conservation, few studies have quantified the relationship among the real microstructures, micromechanical properties, and macroscopic acoustic responses of earthen-site soils. This paper develops a micromechanics-based multiscale model for quantitatively exploring the ultrasonic propagation characteristics of elastic waves in untreated and consolidated earthen-site soils. Scanning electron microscope images and image processing technology are integrated into the finite-element simulation. The effects of microstructure and wave features on the acoustic characteristics of soils are quantitatively investigated under pulsive loading. The simulation results of untreated and consolidated soils are efficiently compared to ultrasonic test data. It is demonstrated that the integration of microstructure image processing and multiscale modeling can predict the ultrasonic pulse velocity well, which improves the accuracy of laboratory testing and field monitoring and better serves the evaluation and implementation of engineering practice in the field of heritage conservation.

## 1. Introduction

Earthen sites with soils as their primary construction materials are historical remains left by human beings in the long course of history. There are various types of earthen sites, including ancient living quarters, caves, ancient tombs, the Great Wall, ancient cities, and cellar furnaces. In China, famous earthen sites include the pit of Terracotta Warriors in Xi’an, Jiaohe Ancient City in Turpan, and Suoyang City in Anxi. The majority of earthen sites in the field experience severe surface weathering. Therefore, experimental attempts have explored the possibility of applying various consolidants to slow down the aging rate of earthen sites.

Three types of ethyl silicate were applied by spraying in the field of Iraq to repair the weathered surfaces of mud-brick walls [1,2]. Ethyl silicate, as a reinforcing material for porous structures, has the advantages of simple operation and favorable permeability [3]. Earthen structures impregnated with alkaline activation exhibited a significant improvement in water resistance and mechanical strength [4]. Previous studies have undertaken laboratory tests using the collapsed blocks of earthen sites and field tests to ensure the authenticity and validity of experimental data to a certain extent. However, both earthen sites located outdoors and collapsed blocks are protected cultural relics, and undertaking experimental research will cause damage to earthen sites. The method of using collapsed blocks for laboratory tests, which was popular in the past few years, would also essentially accelerate the disappearance of earthen ruins. This is contrary to the principle of minimum intervention in the protection of cultural relics. In addition, a lot of samples, equipment, time, and effort are normally required to study the effect of each variable on the properties of soils, and it is difficult to control various factors so that they remain unchanged, thus reducing the accuracy of experimental observations. Hence, experiments alone can no longer meet the needs of scientific research on various cultural relics such as earth sites.

Elastic wave, as a nondestructive testing method, has been widely used in geotechnical, medical, biological, industrial, and other fields [5,6,7,8]. Ultrasound-based methods can detect the internal damage and deterioration of concrete [9,10,11,12] and evaluate its engineering characteristics such as thermal conductivity [13,14,15]. Prassianakis et al. investigated the dynamic modulus of elasticity and the deterioration index of materials through ultrasonic detection [16]. In the field of cultural relic protection, scholars explored the strength and internal structure of soils by ultrasonic pulse velocity [17,18] and estimated the improvement effect of soils [19]. The ultrasonic pulse velocity can reflect the physical and mechanical properties of materials [20,21]. However, the microscopic factors affecting ultrasonic velocity are not clear enough, and it is difficult to study the influences of microscopic parameters and microstructures on ultrasonic pulse velocity by experiments alone.

Yaman et al. [22] indicated that a meso-mechanical model cannot be applied to predict the modulus of single-phase materials, a semi-empirical model was not suitable for predicting ultrasonic pulse velocity, and theoretical models assuming interphases to be spheroidal were able to model mechanical properties of multi-phase composites. Tan et al. [23] established mathematical models for predicting the ultrasonic pulse velocity, rebound value, and compressive strength of concrete. In addition, a microstructural model for understanding the compressibility and shearing of saturated clay [24], a numerical model for exploring the effect of the rotation of the principal stress axis [25], and a second-gradient elastic–plastic model for granular media [26,27,28] were also derived to explain the influence of micro-scale characteristics on macro-scale behaviors. Although the analytical models based on statistics have demonstrated some basic ideas, few analytical solutions are available to predict the acoustic parameters and dynamic elastic characteristics of material owing to irregular shapes, sizes, and distributions of skeleton particles and pores.

Recent years have witnessed the rapid development of computer technology, and various kinds of numerical simulations have already sprung up. They are constantly optimized and upgraded in different industries, and their computational accuracy has made a breakthrough. For example, a finite-element method (FEM) was adopted to quantify the early-stage corrosion of rebars inside reinforced concrete [29]. Yaman et al. [30] also employed the FEM to simulate the stress wave propagation of a heterogeneous concrete model composed of mortar and aggregate and verified its feasibility. The relation between pore characteristics and ultrasonic pulse velocity was investigated based on a finite-element model of porous cellular concrete [31]. Liu et al. [32] established micromechanics-based finite-element models of concrete and revealed the physical mechanisms of the wave modulus of elasticity being higher than the static modulus of elasticity through a half-sine wave and compression testing techniques [33]. However, none of these research efforts attempted to simulate the effect of consolidants on wave propagation. The relationship between micromechanics and macroscopic physical and mechanical properties of the earthen-site soils are not well understood. 

While numerical simulation provides a new solution for studying the changes in acoustic characteristics of earthen-site soils caused by consolidants, such a technique alone lacks credibility. Hence, it should be supplemented with experimental validation, so as to ensure its accuracy, minimize interference in cultural relics, and improve the economy of the research. 

The present study develops a physics-based procedure for studying the ultrasonic pulse propagation of the consolidated earthen-site soils with scanning electron microscopy (SEM) image-based multiscale finite-element method (FEM). This study aims to visualize how microstructural and elastic properties of consolidated soils affect their acoustic behaviors, to quantify the influence of consolidants on ultrasonic pulse propagation, and to predict the acoustic parameters of soils. The current study is among the first efforts to connect microstructures, wave moduli of elasticity and wave propagation by combining FEM and image processing technology. This will provide a reference for parameter selection in future simulations, promote the development of theoretical research, improve the detection accuracy of engineering testing, and better serve evaluation and analysis in engineering practice, thus having a broad application space.

## 2. Materials and Methods

### 2.1. Sample Preparation and Experimental Data

**Figure 1:**
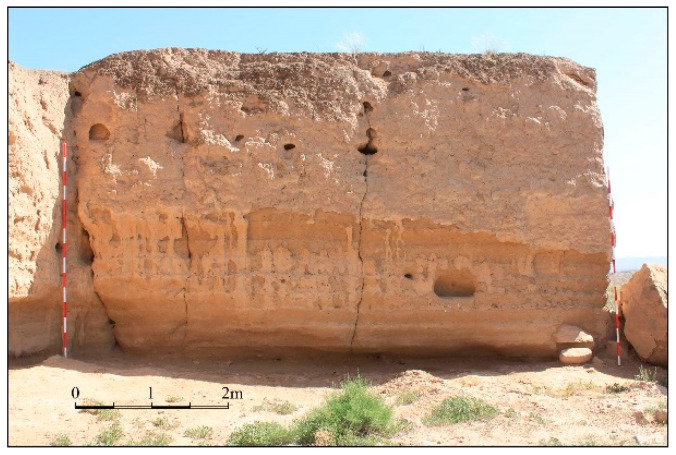
The Great Wall of the Ming Dynasty in Yongchang County, Gansu Province, China.

**Figure 2:**
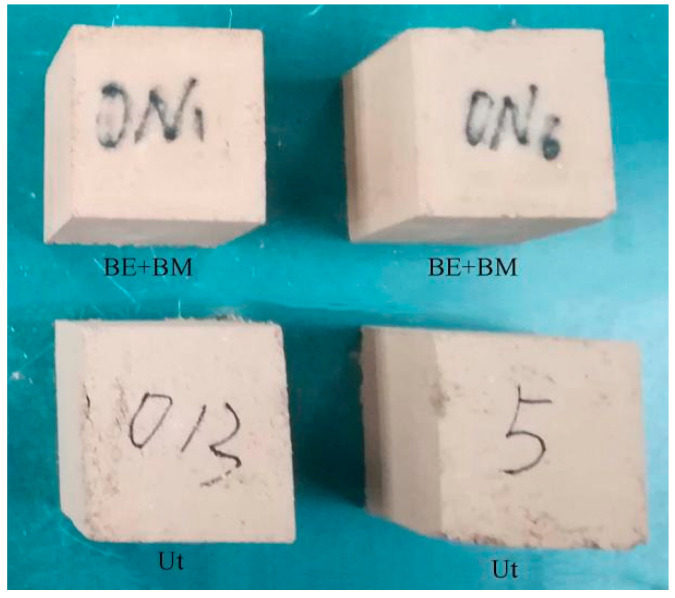
Photographs of the samples.

**Figure 3:**
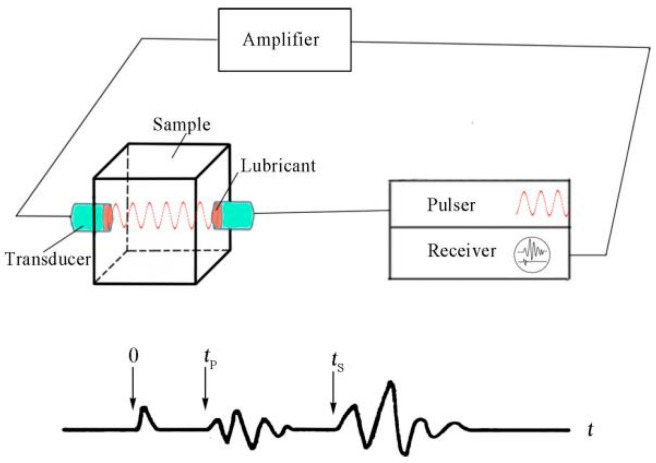
Sound test on the sample.

The soils used in this study were collected from the collapse of the Great Wall of the Ming Dynasty in Yongchang County, Gansu Province, China (Figure 1). The soils were pressed to 50 mm cubic samples after a series of procedures. The samples were treated by Bio Line^®^ Ethyl silicate + Bio Line^®^ Micron lime (BE + BM) after curing for 28 days in laboratory conditions (Figure 2). Bio Line^®^ Ethyl silicate and Bio Line^®^ Micron lime were provided by Shanghai Desaibao Building Materials Co., Ltd., Shanghai, China. The untreated (Ut) samples were used as reference to evaluate the consolidation effect of the BE + BM. Ultrasonic testing equipment with two transducers was applied to acquire the propagation of ultrasound in the soils (Figure 3). The transducers were arranged coaxially on the surfaces of samples. One transducer acted as the pulser to transmit incident waves at a frequency of 50 kHz and the other as the receiver. The ultrasonic wave velocities of Ut and BE + BM samples were 857 m/s and 848 m/s, respectively, in accordance with Chen’s study [34].

### 2.2. Simulation of Ultrasonic Propagation in Soils

#### 2.2.1. Modeling Process and Microstructural Characterization

**Figure 4:**
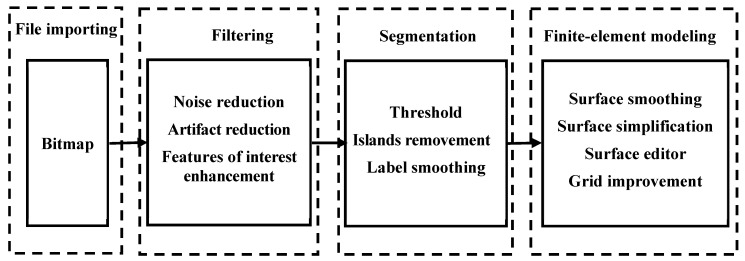
Image processing procedures.

The real microstructures of samples were obtained from the scanning electronic microscopy (SEM) equipment [35]. While a wide range of magnifications are available in SEM tests, the magnification of an SEM image affects, to some extent, the acquisition of soil microstructure in modeling. Images with larger magnifications generally contain fewer particles, which may not be sufficient to represent the real microstructure of soils accurately. However, microstructures of soils at smaller magnifications cannot be clearly displayed, which would cause the study to be meaningless. Therefore, it is important to select a reasonable magnification. The images scanned by SEM were converted into a format acceptable to the finite-element software Marc Mentat 2019 (Figure 4). The SEM image was adjusted to a BMP file and was then preprocessed by using the Nonlocal Means Filter. The filter can correct uneven shadow, weaken the phenomenon of uneven brightness of the image, remove artifacts, reduce noise, improve the quality of the image, strengthen the edge, and improve the contrast between different objects. Subsequently, the threshold segmentation method was used to identify the boundary between different objects based on pixel intensity. Threshold was also called a critical value, which herein refers to the critical value of pixel intensity. Isolated points with small area and little influence on the microstructure of the soil were removed. The number of nodes with little effect on the accuracy of particle morphology was reduced, which greatly improved the computational efficiency of the finite element numerical simulation. Finally, surface smoothing, surface simplification, surface editing, and grid improvement were conducted to obtain the finite element model.

The simulation results of the propagation of simple harmonics in soils were confirmed by wave theory using a square model without interphases. The time of flight (TOF) of a simple harmonic wave can be determined by the time difference between the first peak of input and transmitted signals [32], and then, the velocities of the secondary wave and primary wave can be calculated from the distance between the two transducers and the time of flight (TOF) [36]. The input properties of the square model without interphases included a wave modulus of elasticity of 161 MPa, a mass density of 1.605 g/cm^3^, and a Poisson’s ratio of 0.44. The theoretical values of ultrasonic pulse velocity and dynamic elastic coefficient can be calculated by wave theory for a single medium without interphases and with a regular shape. The reliability of the propagation characteristics of simple harmonic waves in soils can be assessed by comparing the deviation between the finite-element simulation results and the theoretical values.

#### 2.2.2. Representative Volume Element

Earthen-site soils, as an inhomogeneous medium, have complex and diverse particle compositions and structures. In continuum mechanics, constitutive relations are usually applied to study the response of a homogeneous medium to loads. Heterogeneous soils can be equivalent to a homogeneous material, and the representative volume element (RVE) should be selected [37,38]. The RVE must be small enough macroscopically to be regarded as a particle, so that the stress and strain fields are uniform in the RVE, namely,

(1) R<<L

where R is the scale of RVE, and *L* is macroscopic scale.

In addition, the RVE should be large enough and representative from the microscopical perspective to contain rich microstructures, namely,

(2) R>>d

where *d* is the characteristic scale of microstructure.

#### 2.2.3. Ultrasonic Excitation

Since the exciting and receiving positions of simple harmonic waves will affect simulation results, the quantity of transducers should be enough to excite each component of the RVE and receive signals through each part. Therefore, a half-sine pulse with a frequency of 50 kHz was applied to each node on the top boundary of RVE, and the propagation direction of the wave was the same as the vibration direction. The remaining boundaries were free. The transmission signals were received at the opposite side of the incident position of the RVE. The time step was set to 0.05 μs, and the total number of steps was 2000. The number of nodes on an edge of the RVE was 85, which met the requirements of calculation accuracy, saved time, and improved calculation efficiency. For a clearer view of the sinusoidal waveform, the amplitude was set to 1 × 10^5^.

#### 2.2.4. Simulation Parameters

The air-dried soils can be considered as a two-phase material including a solid phase (matrix) and a gaseous phase (pores). The Bio Line^®^ Ethyl silicate + Bio Line^®^ Micron lime (BE + BM) sample can be considered as a three-phase material including two solid phases (matrix and interphase) and a gaseous phase (pores). The parameters required to study the mechanical behavior of soils under the sine pulse can be divided into two categories. One is the physical parameters related to the size, load, and boundary conditions of the RVE. The other is the microscopic dynamic parameters related to the properties of the medium. The mass density, dynamic modulus, and Poisson’s ratio of the material had great influence on the output results of the finite-element simulation, thus affecting the acoustic parameters of soils.

The mass density of soils was obtained from the geotechnical test, noted to be 1.605 g/cm^3^. The Poisson’s ratio of the matrix was 0.44. Previous studies have shown that the static elastic modulus was smaller than the dynamic elastic modulus [39,40,41]. The static elastic modulus of the matrix was 161 MPa [42], and the dynamic elastic modulus of the matrix took 1610 MPa to predict the acoustic characteristics of the soils.

All interphase elements were removed from the Bio Line^®^ Ethyl silicate + Bio Line^®^ Micron lime (BE + BM) model, and only matrix elements were left. In this case, the model was named BE + BM-soil to study the effect of interphases on acoustic parameters. The mass density of the BE + BM-treated sample could be determined from a geotechnical test and was 1.620 g/cm^3^, the dynamic Poisson’s ratio of the matrix and interphase was 0.44, and the dynamic elastic modulus of the matrix was 1610 MPa. Assuming that the dynamic elastic constants of the interphase were consistent with those of the matrix, micromechanics-based finite-element models were established to predict the acoustic characteristics of consolidated and untreated earthen-site soils, and the simulation results were compared with the laboratory tests.

The dynamic elastic modulus of the matrix was successively set as 260 MPa, 460 MPa, 960 MPa, 1360 MPa, and 2060 MPa to explore the relationship between the micro-dynamic constants and the primary wave velocity of the untreated (Ut) sample. The dynamic Poisson’s ratio and mass density of the matrix remained unchanged to reduce the number of tests, which were 0.44 and 1.605 g/cm^3^, respectively.

In the investigation of the relationship between the micro-dynamic elastic modulus and the acoustic characteristics of consolidated earthen-site soils, the dynamic elastic moduli of the matrix and interphase in the BE + BM model were set as 260 MPa, 460 MPa, 960 MPa, 1360 MPa, and 2060 MPa, respectively. Similarly, other parameters remained constant to reduce the number of tests; namely, the dynamic Poisson’s ratio and the mass density of the matrix and interphase were 0.44 and 1.605 g/cm^3^, respectively. In the study of the relationship between the dynamic elastic modulus and primary wave velocity of the matrix, the dynamic elastic modulus of the interphase was 1610 MPa, which was also true for the interphase.

## 3. Results and Discussion

### 3.1. SEM Results and Acquisition of FEM Models

The equivalent diameters of the Ut and BE + BM samples were mostly in the range of 0.12–0.17 mm, and the size of the sample was 5 × 5 × 5 cm^3^; hence, the scale of the RVE needed to meet:

(3) 0.17 mm<<R<<50 mm

The scale of the RVE needed to be smaller than the wavelength [37,43]. Ultrasonic tests showed that the primary wave velocity of the earthen-site soils fluctuated around 850 m/s, and the frequency was 50 kHz; thus, the wavelength was about 17 mm. The scale of the RVE needed to be smaller than 17 mm. Moreover, the time of flight (TOF) of the wave needed to be longer than the waveform time to ensure the accuracy of the simulation. The size of the RVE needed to be larger than 8.5 mm. In accordance with the above-mentioned arguments, the size of the RVE needed to meet

(4) 8.5 mm<<R<<17 mm

The square region was selected as the RVE according to the ratio of the cubic sample. Since the pixel of the SEM image was 960 × 1280, the area with pixel size of 640 × 640 was selected for finite-element modeling after removing the text region in the SEM image, and the corresponding image size was 0.64 mm × 0.64 mm. Therefore, the selection area was magnified to 20 times the size of the RVE, that is, the size of the RVE was 12.8 mm × 12.8 mm.

The harmonic pulse not only affected the stress and strain at the point of application, but also disturbed the surrounding soils. The soils only had small elastic deformation owing to the small energy of the ultrasonic pulse. The soils could return to their original state once removing the pulser; hence, it belonged to the category of small strain.

**Figure 5:**
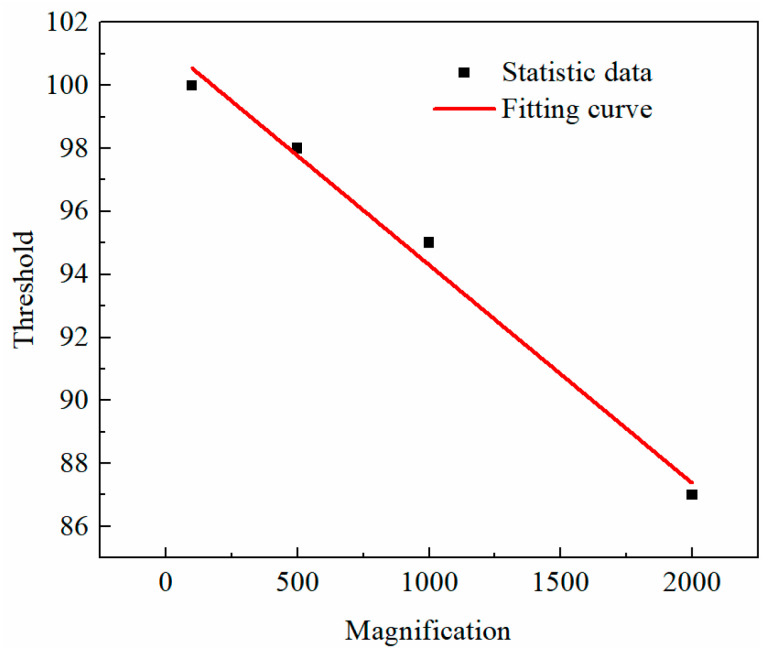
The relationship between magnification and threshold.

**Figure 6:**
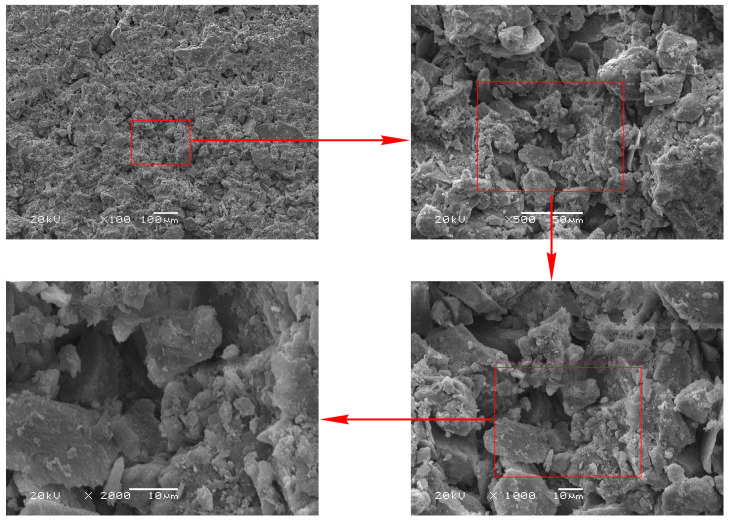
SEM images of the untreated (Ut) sample at different magnifications.

The porosity of the Ut sample was 40.81% [42], and the corresponding threshold decreased with the increase in magnification, indicating that the image with larger magnification contained more pores (Figure 5). As observed in Figure 6, the image magnified 2000 times contained a large pore; thus, it could not accurately represent the microstructure of soils. In addition, 2000-times, 1000-times and 500-times SEM images of the Ut sample had a few particles, and the distribution of pores was obviously uneven. Therefore, the finite-element models of the untreated and consolidated soils were established based on the image with a magnification factor of 100.

**Figure 7:**
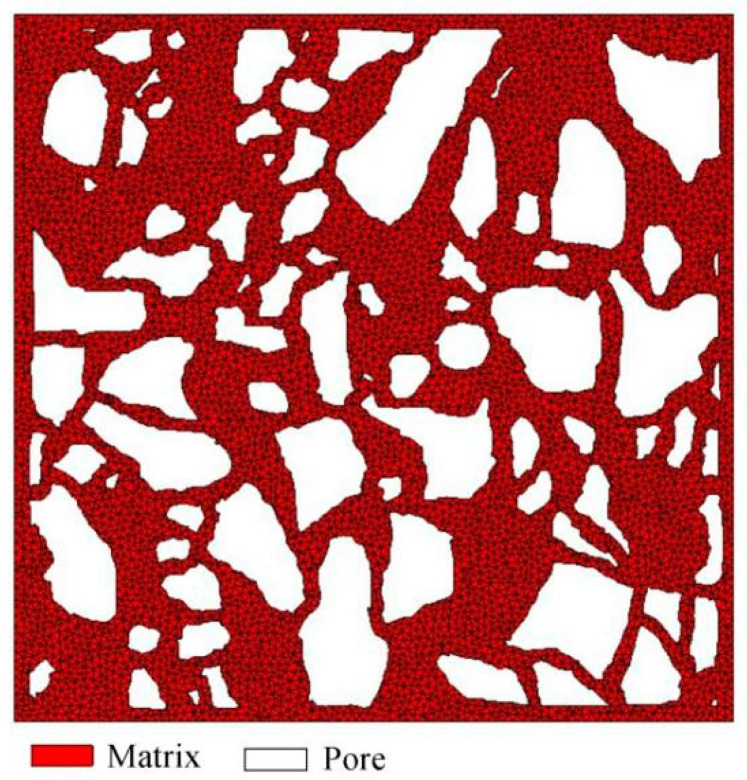
Finite-element model of the untreated (Ut) sample.

**Figure 8:**
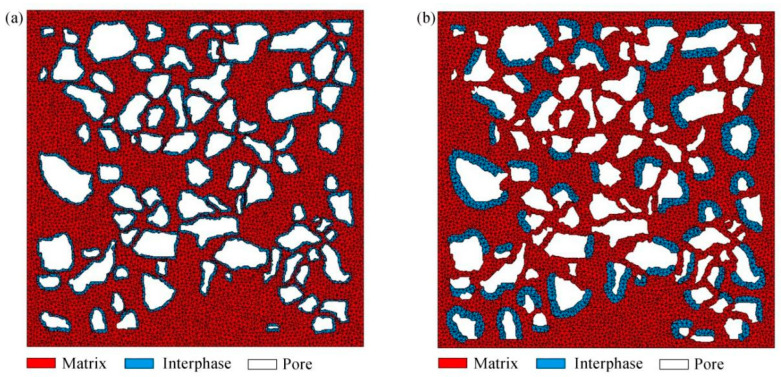
Finite-element models of the Bio Line^®^ Ethyl silicate + Bio Line^®^ Micron lime (BE + BM) samples: (**a**) BE + BM-a; (**b**) BE + BM-b.

The finite-element models of the Ut and Bio Line^®^ Ethyl silicate + Bio Line^®^ Micron lime (BE + BM) consolidated samples were based on the same threshold. The RVE of the Ut sample contained 11,149 triangular elements (Figure 7). Previous studies [44,45] have shown that the deposition of consolidants into samples promotes the growth of strength and reduces the porosity. Nano- and calcium-based consolidants made the connection of mineral particles stronger because of the existence of clay and the increased cementation in the interstitial space [44]. Enzyme-induced carbonate precipitation (EICP) methods shortened the space distance among soil particles, heightened the bonding force, and stimulated the formation of cementitious substances to form a dense structure [45]. It is assumed that there are two ideal distribution modes of consolidants in the microstructures of the consolidated soils. The BE + BM-a model with interphases uniformly distributed on the surface of matrix contained 16,464 triangular elements, while the BE + BM-b model with interphases randomly agglomerated in pores contained 14,682 triangular elements (Figure 8).

### 3.2. Validation of Ultrasonic Simulation-Based Wave Theory

**Figure 9:**
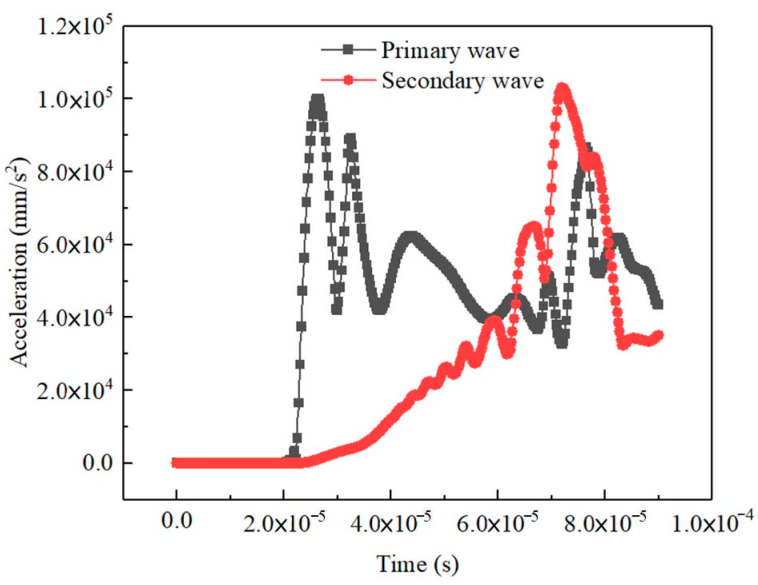
Output signals of the 50 kHz sine incidence in porous medium.

The time of flight (TOF) of the primary wave in the non-porous medium could be obtained from the transmitted signals of the primary wave and secondary wave (Figure 9). The dynamic shear modulus *G_d_* was calculated though the mass density and velocity of the secondary wave:

*G*_*d*_ = *ρ*·*V*_*s*_ (5)

where *G_d_* is the dynamic shear modulus, *ρ* is the mass density, and *V_s_* is the velocity of the secondary wave.

The elastic coefficients can be evaluated from the ultrasonic pulse velocity [36,46,47]. The wave modulus of elasticity and Poisson’s ratio can be estimated by

(6) $$E_{d}=\frac{\rho V_{s}^{2}(3V_{p}^{2}-4V_{s}^{2})}{V_{p}^{2}-V_{s}^{2}}$$

(7) $$\nu_{d}=\frac{V_{p}^{2}-2V_{s}^{2}}{2(V_{p}^{2}-V_{s}^{2})}$$

where *E_d_* is the wave modulus of elasticity, and *V_p_* is the velocity of primary wave, and *ν_d_* is the Poisson’s ratio.

The theoretical value of the dynamic shear modulus of the matrix can be determined by the wave modulus of elasticity and the Poisson’s ratio:

(8) $$G_{d}=\frac{E_{d}}{2+2\nu_{d}}$$

The theoretical values of the velocities of the primary wave and secondary wave are derived from Equations (9) and (10):

(9) $$V_{s}=\sqrt{\frac{E_{d}}{(2+2\nu_{d})\rho }}$$

(10) $$V_{p}=\sqrt{\frac{E_{d}(1-\nu_{d})}{(1+\nu_{d})(1-2\nu_{d})\rho }}$$

The simulated velocities of both the primary wave and secondary wave were slightly higher than the theoretical ones (Table 1). The dynamic elastic parameters also had the same rule. The output results of the finite-element method were generally the same as the theoretical values, and the deviations were less than 6%. Therefore, it was feasible to simulate the propagation of simple harmonics in the medium by the finite-element method.

### 3.3. Micromechanics Models of Untreated Samples for Ultrasonic Pulse Velocity Prediction

**Figure 10:**
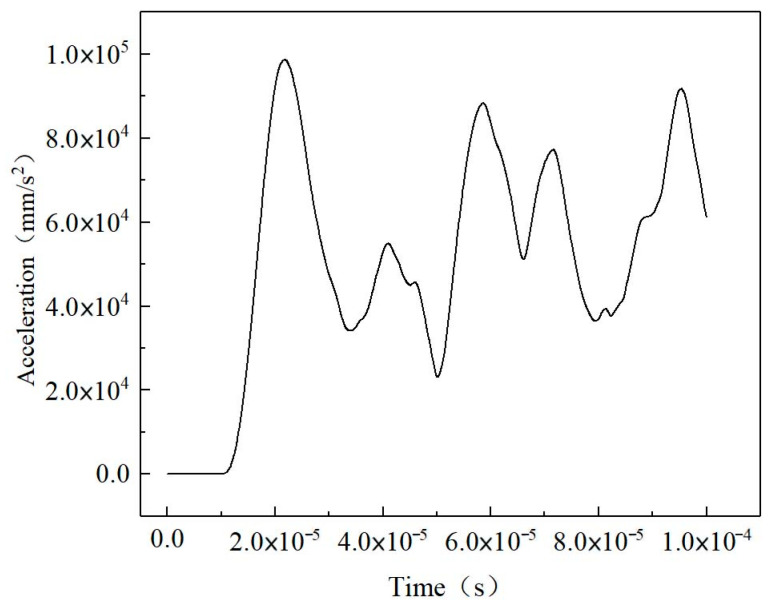
The transmitted signals of the primary wave in the untreated (Ut) sample.

The time interval between the first peak of the transmitted signals and the first peak of the sine incidence was taken as the time of flight (TOF). The output wave reached the first peak at 21.75 μs, indicating that the transmission time of the primary wave in the untreated sample was 16.75 μs (Figure 10). The ultrasonic wave velocity of the Ut sample predicted by the finite-element method was 764.18 m/s, and that measured in the ultrasonic test was 857 m/s. The deviation between the simulated velocity and the experimental value was −10.83%. The value of the wave modulus of elasticity was approximate, and the micromechanics-based finite-element method used to predict the acoustic parameters of the untreated soils was consistent with the experimental data; thus, it was effective to quantitatively explore the propagation characteristics of elastic waves in untreated soils by converting SEM images into finite-element models. The established symmetric model, setup of the simulation, and input microscopic parameters were reasonable. The macroscopic dynamic behaviors of the untreated soils under simple harmonic vibration could be predicted effectively by using the micro-images of soils and reasonable micro-dynamic elastic coefficients.

### 3.4. Micromechanics Models of Consolidated Soils for Ultrasonic Pulse Velocity Prediction

**Figure 11:**
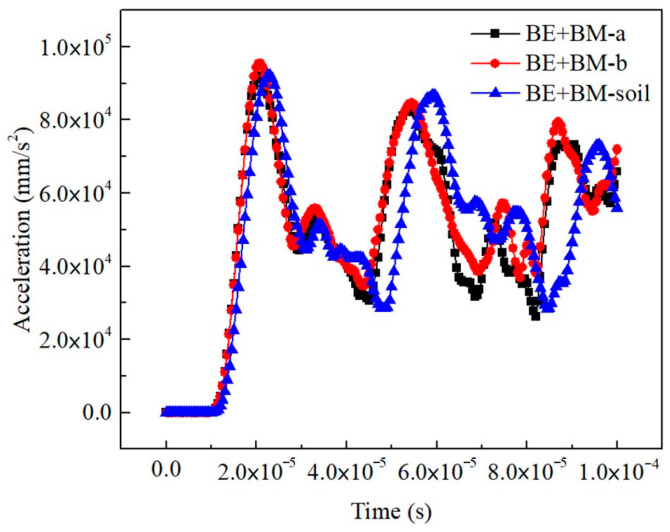
The transmitted signals of the primary wave in the BE + BM sample.

The results of the BE + BM-a model and the BE + BM-b model for ultrasonic pulse velocity (UPV) simulation were similar when the same microscopic input parameters, loads, and boundary conditions were employed. Two curves of the output signals were generally consistent, the deviation between the curves after about 60 μs was due to the difference in the distribution of matrix and interphase in the BE + BM models, and the different interface between two materials resulted in different grid division. While meshes with large aspect ratios were removed during model generation, subtle differences in microstructures contributed to different acoustic responses of the consolidated soils (Figure 11). The grids of the interphase in the BE + BM-a model and the BE + BM-b model were removed, and the porosity of the model, named the BE + BM-soil model, was consistent with that of the untreated sample. According to the output signals of the BE + BM-soil model, the interphases delayed the appearance of the first peak for a period compared with the two consolidated models, and the arrival time of the first peak was approximately 22.75 μs. The primary wave velocity of the sample without interphases was 721 m/s. The consolidants in the BE + BM-a model promoted the ultrasonic wave velocity by 13.42%, and that in the BE + BM-b model caused the primary wave velocity to increase by 13.06%. The existence of interphases accelerated the transmission process of harmonic vibration in soils. The BE + BM-a model and BE + BM-b model had almost the same primary wave velocity, and their simulation results were slightly smaller than that of the ultrasonic test, with absolute deviation of less than 4% (Table 2). Therefore, the simulation results of the BE + BM-a model and the BE + BM-b model were basically the same as the test. The finite-element models, microscopic dynamic parameters, boundary conditions, loading conditions, analysis types, and other settings of the untreated samples were verified by ultrasonic tests, and the prediction results of acoustic parameters of the consolidated soils (BE + BM sample) by FEM were consistent with the experimental data. Therefore, the finite-element modeling based on microscopic images was correct. The acoustic characteristics of the untreated and consolidated soils could be predicted by combining microscopic images, image processing technology, and the finite-element method.

### 3.5. Effect of Micromechanical Parameters on Ultrasonic Characteristics

#### 3.5.1. Effect of Matrix in a Two-Phase Material

**Figure 12:**
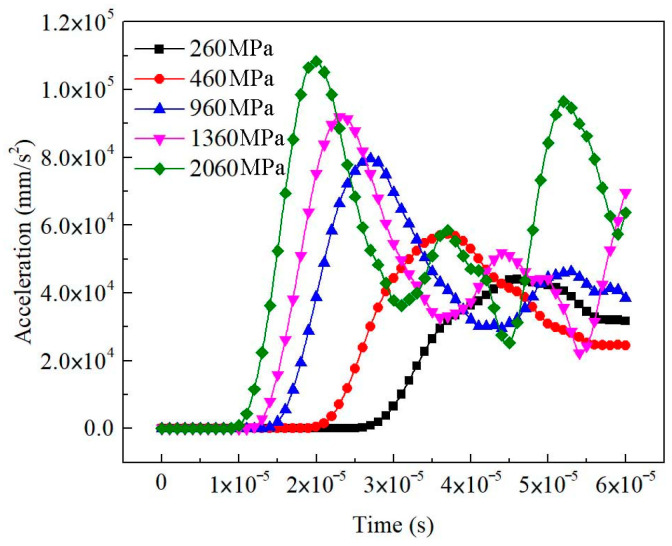
Output waveform of untreated soils with different wave moduli of elasticity.

**Figure 13:**
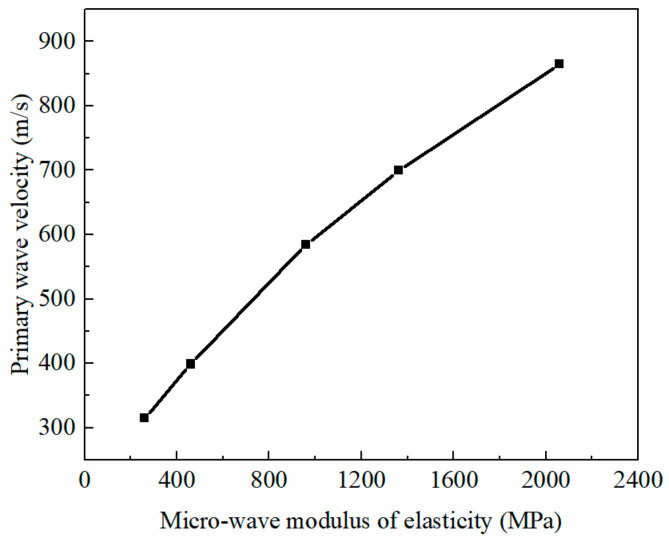
Relationship between wave modulus of elasticity of matrix and primary wave velocity of soils.

The untreated sample, as a two-phase material, included a solid phase (matrix) and a gaseous phase (pores). When the wave modulus of the elasticity of the matrix was set to a series of different values, the macroscopic waveform curves of soils had the same trend and showed obvious regularity (Figure 12). The receiving position started to vibrate earlier and earlier with the increase in the wave modulus of the elasticity of the matrix, and the first peak value of macroscopic acceleration increased gradually. For porous earthen-site soils, the primary wave velocity was directly proportional to the wave modulus of the elasticity of the matrix (Figure 13). The experimental value of the ultrasonic pulse velocity of the untreated sample was 857 m/s, and the wave modulus of the elasticity of the matrix could be calculated, according to the fitting relation between the wave modulus of the elasticity of the matrix and the primary wave velocity, to be 1957 MPa. The static elastic modulus of the matrix was 161 MPa. Hence, the wave modulus of the elasticity of the matrix was much larger than the static elastic modulus, which was about 12 times the size of the static elastic modulus.

#### 3.5.2. Effect of Matrix and Interphases in Three-Phase Materials

**Figure 14:**
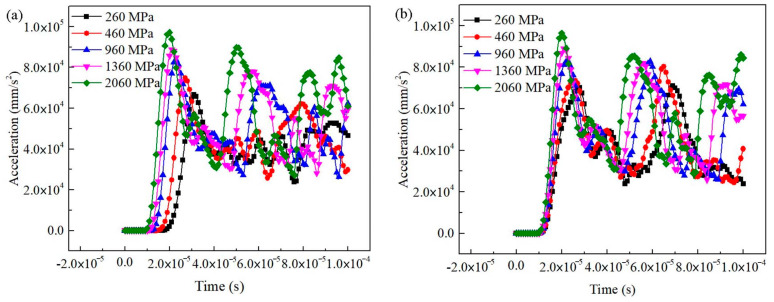
Effect of wave modulus of elasticity of matrix and interphase on output waveform in BE + BM-a model: (**a**) matrix; (**b**) interphase.

The Bio Line^®^ Ethyl silicate + Bio Line^®^ Micron lime (BE + BM) sample, as a three-phase material, included two solid phases (matrix and interphase) and a gaseous phase (pores). For the BE + BM-a model, the wave moduli of elasticity of matrix and interphase both had certain influence on the output signals (Figure 14). After a pulse emission of 50 μs, the waveforms with different wave moduli of the matrix had no obvious regularity due to the large change, while the received signals with different wave moduli of interphases had regular changes. The change in the wave modulus of the elasticity of the matrix had a slightly greater effect on the waveform than that of the interphase.

**Figure 15:**
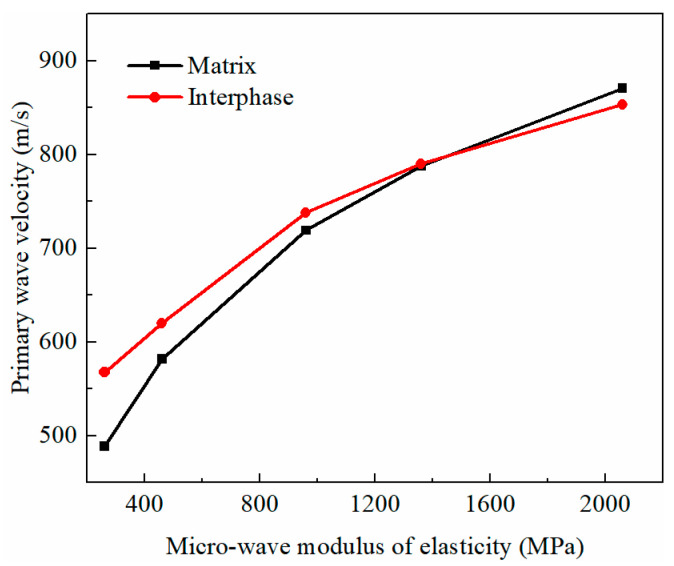
The relation between wave modulus of elasticity and primary wave velocity in BE + BM-a model.

In the BE + BM-a model, when the wave moduli of the elasticity of the matrix and interphase changed from low to high, the ultrasonic wave velocity of the consolidated soils changed dramatically (Figure 15). Compared with the higher wave modulus of elasticity, the ultrasonic pulse velocity was more sensitive to the wave moduli of the matrix and interphase in the lower wave modulus range. The wave velocity increased with the increase in the wave moduli of the elasticity of the matrix and interphase. When the wave modulus of the elasticity of the matrix changed, that of the interphase remained unchanged at 1610 MPa; on the contrary, when the wave modulus of the elasticity of the interphase changed, that of the matrix also remained at 1610 MPa. Therefore, the curves of the relationship between the primary wave velocity and the wave modulus of the elasticity of the matrix and interphases would intersect at 1610 MPa. When the wave modulus of the elasticity of the matrix increased from 260 MPa to 2060 MPa, the propagation velocity of the primary wave increased by approximately 78%, and when the wave modulus of the elasticity of the interphase increased from 260 MPa to 2060 MPa, the velocity increased by about 50%. The sensitivity of primary wave velocity to the wave modulus of the elasticity of the matrix was slightly stronger than that of the interphase as per the slope of the linear fitting curve of primary wave velocity and the micro-wave modulus of elasticity.

**Figure 16:**
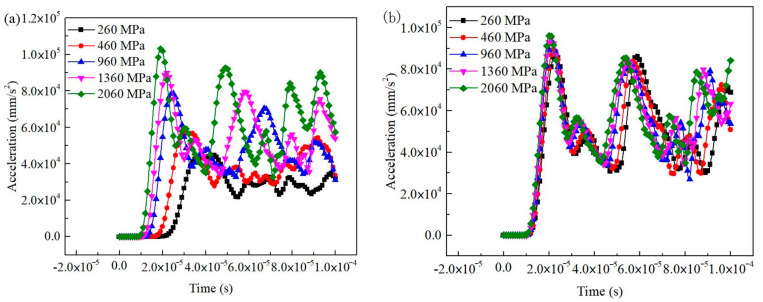
Influence of wave modulus of elasticity of matrix and interphase on output waveform in BE + BM-b model: (**a**) matrix; (**b**) interphase.

For the BE + BM-b model, the wave modulus of the elasticity of the matrix had a significant influence on output signals (Figure 16a). The initial vibration time of the receiving position was obviously different when the wave modulus of the elasticity of the matrix changed. The time of the initial vibration and first peak of the signals were both advanced for a period with the increase in the dynamic elastic modulus of the matrix, indicating that the propagation velocity of the sine wave was accelerated. Similar to the BE + BM-a model, the waveform also became chaotic after 50 μs. However, the signals received at the receiving position were generally consistent when the wave modulus of the elasticity of the interphase changed (Figure 16b). The initial vibration times of the output signals were almost identical, and the acceleration values of the first peak were equal and corresponded to the same time. The output signals were insensitive to the wave modulus of the elasticity of interphases during the receiving period (0–100 μs).

**Figure 17:**
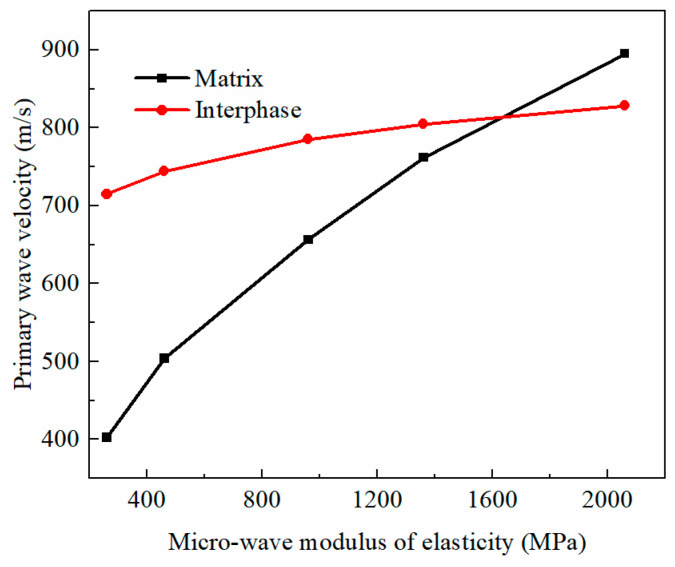
Relationship between wave modulus of elasticity and primary wave velocity in BE + BM-b model.

The primary wave velocity had a positive linear correlation with the wave modulus of the elasticity of the matrix in the BE + BM-b model (Figure 17), and its slope was large. This demonstrates that the transmission velocity of the sine wave was sensitive to the wave modulus of the elasticity of the matrix. The sensitivity of primary wave velocity to the wave modulus of the elasticity of the matrix and interphase varied greatly. The propagation velocity of the sine wave increased by 122% when the wave modulus of the matrix increased from 260 MPa to 2060 MPa, while the ultrasonic pulse velocity increased by less than 16% when the wave modulus of the elasticity of the interphase rose from 260 MPa to 2060 MPa. According to the slopes of the fitting curves between the ultrasonic pulse velocity and the micro-wave modulus of elasticity, the wave modulus of the elasticity of the matrix in the BE + BM-b model had the greatest influence on the transmission velocity of primary waves, followed by that of the matrix and interphase in the BE + BM-a model. The wave modulus of the elasticity of the interphase in the BE + BM-b model had the least effect on the transmission velocity of the sine pulse.

## 4. Conclusions

This study developed a micromechanics-based multiscale model to quantify the influence of the wave modulus of elasticity and microstructure characteristics on the acoustic parameters of untreated and consolidated soils. The main conclusions can be drawn as follows:

1. The predicted acoustic characteristics of untreated and consolidated earthen-site soils agree well with the experimental results. It is feasible to convert SEM images into the finite-element model by image processing technology to quantify the propagation characteristics of simple harmonic vibration in earthen-site soils. The acoustic characteristics of soils can be predicted effectively by using microstructural images and reasonable micro-dynamic elastic coefficients.
2. For porous earthen-site soils, the primary wave velocity is directly proportional to the wave modulus of the elasticity of the matrix. The microscopic wave modulus of the elasticity of earthen-site soils is much larger than the static elastic modulus, which is about 12 times that of the static elastic modulus.
3. For the consolidated-soil model with interphases uniformly surrounding the surface of the matrix, the output waveform of the sine pulse is slightly more sensitive to the wave modulus of the elasticity of the matrix than that of interphase. For the consolidated-soil model with interphases randomly agglomerated in pores, the output waveform is sensitive to the wave modulus of the elasticity of the matrix, but very insensitive to that of the interphase. The wave modulus of the elasticity of the matrix in the random agglomeration consolidated-soil model has the greatest influence on ultrasonic pulse velocity, followed by the wave moduli of the elasticity of the matrix and interphase in the uniform consolidated-soil model, and the wave modulus of the elasticity of the interphase in the random agglomeration consolidated-soil model has the least influence on the propagation velocity of the sine pulse.
4. A micromechanics-based finite element method is proposed to explore the propagation process of harmonic pulses in soils. Quantitative understanding of acoustic response characteristics is helpful to improve the accuracy of the ultrasonic detection of earthen-site soils.

## Figures and Tables

**Table 1:** Comparison of theoretical and simulated values.

	*V_p_* (m/s)	*V_s_* (m/s)	*G_d_* (MPa)	*ν_d_*	*E_d_* (MPa)
Theoretical value	570.16	186.63	55.90	0.440	161.00
Simulated value	599.53	190.90	58.49	0.444	168.88
Deviation (%)	5.15	2.29	4.63	0.81	4.89

**Table 2:** Comparison of ultrasonic pulse velocities between simulated and experimental data.

Experimental Value (m/s)	BE + BM-a Model		BE + BM-b Model	
	Simulated Value (m/s)	Error (%)	Simulated Value (m/s)	Error (%)
848	818	3.55	815	3.86

## Data Availability

The data used to support the findings of this study are available from the corresponding authors upon reasonable request.
